# 2-Methyl-5-nitro­benzonitrile

**DOI:** 10.1107/S1600536808010982

**Published:** 2008-05-03

**Authors:** Wen-Xian Liang, Guo-Xi Wang

**Affiliations:** a Ordered Matter Science Research Center, College of Chemistry and Chemical Engineering, Southeast University, Nanjing 210096, People’s Republic of China

## Abstract

In the title compound, C_8_H_6_N_2_O_2_, the nitro group is rotated by 10.2 (2)° out of the plane of the benzene ring. The crystal structure is stabilized by van der Waals inter­actions.

## Related literature

For the chemistry of nitrile derivatives, see: Xiong *et al.* (2002 ▶); Jin *et al.* (1994 ▶); Brewis *et al.* (2003 ▶); Dunica *et al.* (1991 ▶). For related literature, see: Fu & Zhao (2007 ▶).
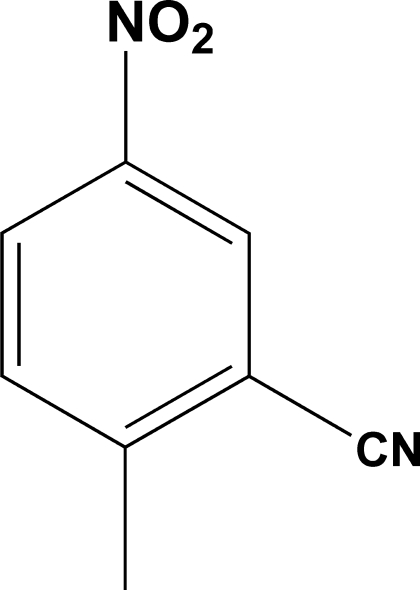


## Experimental

### 

#### Crystal data

C_8_H_6_N_2_O_2_
*M* *_r_* = 162.15
Monoclinic, 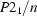

*a* = 3.8946 (8) Å
*b* = 7.6350 (15) Å
*c* = 26.180 (5) Å
β = 91.65 (3)°
*V* = 778.1 (3) Å^3^
*Z* = 4
Mo *K*α radiation
μ = 0.10 mm^−1^
*T* = 293 (2) K
0.4 × 0.35 × 0.2 mm

#### Data collection

Rigaku Mercury2 diffractometer
Absorption correction: multi-scan (*CrystalClear*; Rigaku/MSC, 2005 ▶) *T*_min_ = 0.93, *T*_max_ = 0.98
7390 measured reflections
1761 independent reflections
1273 reflections with *I* > 2σ(*I*)
*R*_int_ = 0.038

#### Refinement

*R*[*F* ^2^ > 2σ(*F* ^2^)] = 0.050
*wR*(*F* ^2^) = 0.141
*S* = 1.04
1761 reflections
109 parameters
H-atom parameters constrained
Δρ_max_ = 0.14 e Å^−3^
Δρ_min_ = −0.18 e Å^−3^

### 

Data collection: *CrystalClear* (Rigaku/MSC, 2005 ▶); cell refinement: *CrystalClear*; data reduction: *CrystalClear*; program(s) used to solve structure: *SHELXS97* (Sheldrick, 2008 ▶); program(s) used to refine structure: *SHELXL97* (Sheldrick, 2008 ▶); molecular graphics: *SHELXTL* (Sheldrick, 2008 ▶); software used to prepare material for publication: *SHELXTL*.

## Supplementary Material

Crystal structure: contains datablocks I, global. DOI: 10.1107/S1600536808010982/bx2137sup1.cif

Structure factors: contains datablocks I. DOI: 10.1107/S1600536808010982/bx2137Isup2.hkl

Additional supplementary materials:  crystallographic information; 3D view; checkCIF report

## Figures and Tables

**Fig. 1.**
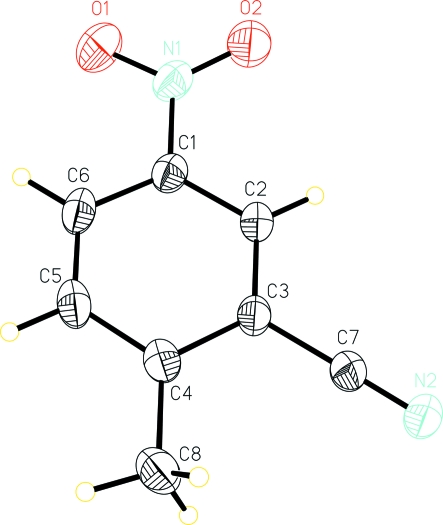
A view of the title compound with the atomic numbering scheme. Displacement ellipsoids were drawn at the 30% probability level.

## supplementary crystallographic information

### Comment

Nitrile derivatives have found wide range of applications in industry and coordination chemistry as ligands. For example, phthalonitriles have been used as starting materials for phthalocyanines (Jin *et al.*, 1994), which are important components for dyes, pigments, gas sensors, optical limiters and liquid crystals, and which are also used in medicine, as singlet oxygen photosensitisers for photodynamic therapy (Brewis *et al.*, 2003). And nitrile compounds are the precursor of tetrazole complexes (Dunica *et al., *1991), which we have focused on for the design of noncentrosymmetric bulk materials, based on axial-chiral ligand 5-(3-methyl-4-nitrophenyl)-2*H*-tetrazole (Xiong *et al., *2002). Recently, we have reported a few benzonitrile compounds (Fu & Zhao, 2007). As an extension of our work on the structural characterization, we report here the crystal structure of title compound. The crystal data show that in the title compound, the benzene ring and the nitro group are nearly planar, they are only twisted to each other by a torsion angles of O2—N1—C1—C2 (-10.4 (2)°) and O1—N1—C1—C6 (-9.9 (2)°), the nitrile group C7—N2 bond length of 1.137 (2)Å is within the normal range (Fig.1).

### Experimental

The  title compound was purchased  from Aldrich and  was dissolved (3 mmol, 486.44 mg)  in ethanol (20 ml) and evaporated in air affording colorless block crystals  suitable for X-ray analysis.

### Refinement

Positional parameters of all the H atoms bonded to C atoms were calculated geometrically and were allowed to ride on the C atoms to which they are bonded, with C—H = 0.93Å (aromatic) and with U_iso_(H) = 1.2_eq_(C) or 0.96Å (methyl) and *U*_iso_(H) = 1.5U_eq_(C).

### Crystal data

**Table:**

C_8_H_6_N_2_O_2_	*F*_000_ = 336
*M**_r_* = 162.15	*D*_x_ = 1.384 Mg m^−^^3^
Monoclinic, *P*2_1_/*n*	Melting point = 349–350 K
Hall symbol: -P 2yn	Mo *K*α radiation λ = 0.71073 Å
*a* = 3.8946 (8) Å	Cell parameters from 1763 reflections
*b* = 7.6350 (15) Å	θ = 3.1–27.7º
*c* = 26.180 (5) Å	µ = 0.10 mm^−^^1^
β = 91.65 (3)º	*T* = 293 (2) K
*V* = 778.1 (3) Å^3^	Block, colourless
*Z* = 4	0.4 × 0.35 × 0.2 mm

### Data collection

**Table:**

Rigaku Mercury2 diffractometer	1761 independent reflections
Radiation source: fine-focus sealed tube	1273 reflections with *I* > 2σ(*I*)
Monochromator: graphite	*R*_int_ = 0.039
Detector resolution: 13.6612 pixels mm^-1^	θ_max_ = 27.5º
*T* = 293(2) K	θ_min_ = 3.1º
ω scans	*h* = −5→4
Absorption correction: multi-scan(CrystalClear; Rigaku/MSC, 2005)	*k* = −9→9
*T*_min_ = 0.93, *T*_max_ = 0.98	*l* = −33→33
7390 measured reflections	

### Refinement

**Table:**

Refinement on *F*^2^	Secondary atom site location: difference Fourier map
Least-squares matrix: full	Hydrogen site location: inferred from neighbouring sites
*R*[*F*^2^ > 2σ(*F*^2^)] = 0.050	H-atom parameters constrained
*wR*(*F*^2^) = 0.141	*w* = 1/[σ^2^(*F*_o_^2^) + (0.0668*P*)^2^ + 0.1332*P*] where *P* = (*F*_o_^2^ + 2*F*_c_^2^)/3
*S* = 1.04	(Δ/σ)_max_ < 0.001
1761 reflections	Δρ_max_ = 0.14 e Å^−^^3^
109 parameters	Δρ_min_ = −0.18 e Å^−^^3^
Primary atom site location: structure-invariant direct methods	Extinction correction: none

### Special details

**Table:**

Geometry. All e.s.d.'s (except the e.s.d. in the dihedral angle between two l.s. planes) are estimated using the full covariance matrix. The cell e.s.d.'s are taken into account individually in the estimation of e.s.d.'s in distances, angles and torsion angles; correlations between e.s.d.'s in cell parameters are only used when they are defined by crystal symmetry. An approximate (isotropic) treatment of cell e.s.d.'s is used for estimating e.s.d.'s involving l.s. planes.
Refinement. Refinement of *F*^2^ against ALL reflections. The weighted *R*-factor *wR* and goodness of fit *S* are based on *F*^2^, conventional *R*-factors *R* are based on *F*, with *F* set to zero for negative *F*^2^. The threshold expression of *F*^2^ > σ(*F*^2^) is used only for calculating *R*-factors(gt) *etc*. and is not relevant to the choice of reflections for refinement. *R*-factors based on *F*^2^ are statistically about twice as large as those based on *F*, and *R*- factors based on ALL data will be even larger.

### Fractional atomic coordinates and isotropic or equivalent isotropic displacement parameters (Å^2^)

**Table:**

	*x*	*y*	*z*	*U*_iso_*/*U*_eq_	
N1	0.8171 (4)	0.3298 (2)	0.18614 (5)	0.0590 (4)	
C3	0.8617 (4)	0.6612 (2)	0.08273 (6)	0.0450 (4)	
C2	0.7935 (4)	0.5051 (2)	0.10776 (5)	0.0451 (4)	
H2A	0.6877	0.4120	0.0907	0.054*	
C7	0.7618 (5)	0.6787 (2)	0.02938 (6)	0.0534 (4)	
C4	1.0183 (4)	0.8045 (2)	0.10793 (6)	0.0489 (4)	
C1	0.8882 (4)	0.4929 (2)	0.15883 (6)	0.0469 (4)	
N2	0.6808 (5)	0.6958 (2)	−0.01239 (6)	0.0745 (5)	
C5	1.1068 (4)	0.7835 (2)	0.15941 (7)	0.0569 (5)	
H5A	1.2101	0.8762	0.1770	0.068*	
O1	0.9413 (5)	0.3114 (2)	0.22899 (6)	0.0954 (6)	
O2	0.6345 (4)	0.2205 (2)	0.16517 (6)	0.0838 (5)	
C6	1.0461 (4)	0.6299 (2)	0.18501 (6)	0.0550 (5)	
H6A	1.1100	0.6182	0.2193	0.066*	
C8	1.0878 (5)	0.9721 (2)	0.08010 (7)	0.0636 (5)	
H8A	1.1998	1.0535	0.1030	0.095*	
H8B	0.8748	1.0213	0.0676	0.095*	
H8C	1.2333	0.9487	0.0519	0.095*	

### Atomic displacement parameters (Å^2^)

**Table:**

	*U*^11^	*U*^22^	*U*^33^	*U*^12^	*U*^13^	*U*^23^
N1	0.0639 (9)	0.0679 (10)	0.0450 (8)	0.0035 (8)	−0.0017 (7)	0.0075 (7)
C3	0.0453 (8)	0.0507 (9)	0.0387 (8)	−0.0026 (7)	−0.0039 (6)	−0.0050 (6)
C2	0.0460 (8)	0.0481 (9)	0.0407 (8)	−0.0017 (7)	−0.0045 (6)	−0.0051 (6)
C7	0.0660 (11)	0.0465 (9)	0.0473 (9)	−0.0131 (8)	−0.0079 (8)	−0.0006 (7)
C4	0.0438 (8)	0.0519 (9)	0.0509 (9)	−0.0024 (7)	−0.0011 (7)	−0.0091 (7)
C1	0.0445 (8)	0.0559 (10)	0.0401 (8)	0.0037 (7)	−0.0011 (6)	−0.0006 (7)
N2	0.1069 (14)	0.0667 (10)	0.0486 (9)	−0.0268 (9)	−0.0196 (9)	0.0072 (7)
C5	0.0547 (10)	0.0629 (11)	0.0525 (10)	−0.0062 (8)	−0.0072 (8)	−0.0186 (8)
O1	0.1169 (13)	0.1119 (13)	0.0559 (8)	−0.0133 (10)	−0.0234 (8)	0.0294 (8)
O2	0.1135 (13)	0.0681 (9)	0.0688 (9)	−0.0226 (9)	−0.0121 (9)	0.0108 (7)
C6	0.0540 (10)	0.0721 (12)	0.0385 (8)	0.0022 (8)	−0.0078 (7)	−0.0093 (7)
C8	0.0640 (11)	0.0545 (10)	0.0721 (12)	−0.0136 (9)	−0.0039 (9)	−0.0046 (9)

### Geometric parameters (Å, °)

**Table:**

N1—O2	1.217 (2)	C4—C5	1.391 (2)
N1—O1	1.2168 (19)	C4—C8	1.501 (2)
N1—C1	1.467 (2)	C1—C6	1.385 (2)
C3—C4	1.407 (2)	C5—C6	1.375 (3)
C3—C2	1.390 (2)	C5—H5A	0.9300
C3—C7	1.445 (2)	C6—H6A	0.9300
C2—C1	1.380 (2)	C8—H8A	0.9600
C2—H2A	0.9300	C8—H8B	0.9600
C7—N2	1.137 (2)	C8—H8C	0.9600
			
O2—N1—O1	123.27 (16)	C2—C1—N1	118.73 (14)
O2—N1—C1	118.58 (14)	C6—C1—N1	119.18 (14)
O1—N1—C1	118.15 (15)	C6—C5—C4	121.98 (15)
C4—C3—C2	122.14 (14)	C6—C5—H5A	119.0
C4—C3—C7	118.83 (14)	C4—C5—H5A	119.0
C2—C3—C7	119.02 (13)	C5—C6—C1	118.89 (15)
C1—C2—C3	117.73 (14)	C5—C6—H6A	120.6
C1—C2—H2A	121.1	C1—C6—H6A	120.6
C3—C2—H2A	121.1	C4—C8—H8A	109.5
N2—C7—C3	178.63 (19)	C4—C8—H8B	109.5
C5—C4—C3	117.16 (15)	H8A—C8—H8B	109.5
C5—C4—C8	121.71 (15)	C4—C8—H8C	109.5
C3—C4—C8	121.12 (15)	H8A—C8—H8C	109.5
C2—C1—C6	122.09 (15)	H8B—C8—H8C	109.5
			
C4—C3—C2—C1	−0.7 (2)	O1—N1—C1—C2	170.40 (16)
C7—C3—C2—C1	−179.26 (14)	O2—N1—C1—C6	169.28 (16)
C2—C3—C4—C5	0.6 (2)	O1—N1—C1—C6	−9.9 (2)
C7—C3—C4—C5	179.19 (15)	C3—C4—C5—C6	0.2 (2)
C2—C3—C4—C8	−179.73 (15)	C8—C4—C5—C6	−179.50 (16)
C7—C3—C4—C8	−1.1 (2)	C4—C5—C6—C1	−0.8 (3)
C3—C2—C1—C6	0.0 (2)	C2—C1—C6—C5	0.7 (3)
C3—C2—C1—N1	179.64 (13)	N1—C1—C6—C5	−178.91 (15)
O2—N1—C1—C2	−10.4 (2)		
